# Beyond weight loss: a review of the therapeutic uses of very-low-carbohydrate (ketogenic) diets

**DOI:** 10.1038/ejcn.2013.116

**Published:** 2013-06-26

**Authors:** A Paoli, A Rubini, J S Volek, K A Grimaldi

**Affiliations:** 1 The Physiological Laboratory, Department of Biomedical Sciences, University of Padova, Padova, Italy; 2 Human Performance Laboratory, Department of Kinesiology, University of Connecticut, Storrs, CT, USA; 3 Biomedical Engineering Laboratory, Institute of Communication and Computer Systems, National Technical University of Athens, Athens, Greece

**Keywords:** ketogenic diet, cancer, diabetes, neurological diseases, obesity, cardiovascular diseases

## Abstract

Very-low-carbohydrate diets or ketogenic diets have been in use since the 1920s as a therapy for epilepsy and can, in some cases, completely remove the need for medication. From the 1960s onwards they have become widely known as one of the most common methods for obesity treatment. Recent work over the last decade or so has provided evidence of the therapeutic potential of ketogenic diets in many pathological conditions, such as diabetes, polycystic ovary syndrome, acne, neurological diseases, cancer and the amelioration of respiratory and cardiovascular disease risk factors. The possibility that modifying food intake can be useful for reducing or eliminating pharmaceutical methods of treatment, which are often lifelong with significant side effects, calls for serious investigation. This review revisits the meaning of physiological ketosis in the light of this evidence and considers possible mechanisms for the therapeutic actions of the ketogenic diet on different diseases. The present review also questions whether there are still some preconceived ideas about ketogenic diets, which may be presenting unnecessary barriers to their use as therapeutic tools in the physician's hand.

## Introduction

During recent years, an increasing amount of evidence has accumulated in the literature, suggesting that very-low-carbohydrate ketogenic diets (VLCKD) could have a therapeutic role in numerous diseases. The use of VLCKD in treating epilepsy has been well established for many decades and these diets have become even more widely known, as they became popular in the 1970s for weight loss—especially as the ‘Atkins Diet'.^1^ More recently, the therapeutic use of ketogenic diets in other diseases has been studied with positive results—it is an important direction for research because, clearly, if nutritional intervention can reduce reliance on pharmaceutical treatments it would bring significant benefits from an economic as well as a social point of view given the current US $750 billion annual cost of pharmaceuticals.^2^

Ketogenic diets are characterized by a reduction in carbohydrates (usually to less than 50 g/day) and a relative increase in the proportions of protein and fat.^3^ The knowledge regarding the metabolic effects of classic ketogenic diets originates from the pioneering work of Cahill and colleagues in the 1960s,^4^ but the realization of the importance of these diets from a clinical point of view can be traced back to the early 1920s when they began to be successfully used in the treatment of epilepsy.^5^ There even appears to be a reference to its use in the Bible in the story of the cured epileptic (New Testament, Matthew 17:14–21). Alongside the huge amount of data about the influence of correct nutrition on health status and disease prevention (encapsulated in various nutritional guidelines delivered by public health committees worldwide), there is also ample evidence to support the notion that a low-carbohydrate diet can lead to an improvement in some metabolic pathways and have beneficial health effects. To use ‘food as medicine' is as attractive a concept as it is ancient, and in the hope of realizing this much effort has been dedicated to exploring the effects of VLCKD on human metabolism. In this review we will look at all the areas where ketogenic diets have been proposed as having potential clinical utility with a brief discussion of the evidence.

## What is ketosis?

Insulin activates key enzymes in pathways, which store energy derived from carbohydrates, and when there is an absence or scarcity of dietary carbohydrates the resulting reduced insulin level leads to a reduction in lipogenesis and fat accumulation. After a few days of fasting, or of drastically reduced carbohydrate consumption (below 50 g/day), glucose reserves become insufficient both for normal fat oxidation via the supply of oxaloacetate in the Krebs cycle (which gave origin to the phrase ‘fat burns in the flame of carbohydrate') and for the supply of glucose to the central nervous system (CNS).^4^

The CNS cannot use fat as an energy source; hence, it normally utilizes glucose. After 3–4 days without carbohydrate consumption the CNS is ‘forced' to find alternative energy sources, and as demonstrated by the classic experiments of Cahill and colleagues^4^ this alternative energy source is derived from the overproduction of acetyl coenzyme A (CoA). This condition seen in prolonged fasting, type 1 diabetes and high-fat/low-carbohydrate diets leads to the production of higher-than-normal levels of so-called ketone bodies (KBs), that is, acetoacetate, β-hydroxybutyric acid and acetone—a process called ketogenesis and which occurs principally in the mitochondrial matrix in the liver.^6^

The main KB produced in the liver is acetoacetate but the primary circulating ketone is β-hydroxybutyrate although the latter is not, strictly speaking, a KB because the ketone moiety has been reduced to a hydroxyl group. Under normal conditions of adequate dietary carbohydrate, the production of free acetoacetic acid is negligible and it is rapidly metabolized by various tissues, especially the skeletal and heart muscles. In conditions of overproduction of acetoacetic acid, it accumulates above normal levels and part of it is converted to the other two KBs leading to ketonemia and ketonuria (presence of KBs in the blood and urine). The characteristic ‘sweet' breath odour of ketosis is caused by acetone, which, being a very volatile compound, is eliminated mainly via respiration in the lungs. The pathway that results in the formation of 3-hydroxy-3-methylglutaryl–CoA from acetyl CoA also occurs in the cytosol of hepatic cells where it is used instead for the biosynthesis of cholesterol. Under normal conditions, the concentration of KBs is very low (<0.3 mmol/l) compared with glucose (∼4 mmol), and as glucose and KBs have a similar kM for glucose transport to the brain the KBs begin to be utilized as an energy source by the CNS when they reach a concentration of about 4 mmol/l, which is close to the Km for the monocarboxylate transporter.^3, 6^

KBs are then used by tissues as a source of energy^3^ through a pathway that leads to formation from β-hydroxybutyrate of two molecules of acetyl CoA, which are used finally in the Krebs cycle. It is interesting to note that the KBs are able to produce more energy compared with glucose because of the metabolic effects of ketosis—the high chemical potential of 3-β-hydroxybutyrate leads to an increase in the Δ*G*_0_ of ATP hydrolysis.^3^ A further point to underline is, as shown in Table 1, that glycaemia, even though reduced, remains within physiological levels because of the fact that glucose is formed from two sources: from glucogenic amino acids and from glycerol liberated via lysis from triglycerides.^7^

We would like to emphasize that ketosis is a completely physiological mechanism and it was the biochemist Hans Krebs who first referred to physiological ketosis to differentiate it from the pathological keto acidosis seen in type 1 diabetes.^8^ In physiological ketosis (which occurs during very-low-calorie ketogenic diets), ketonemia reaches maximum levels of 7/8 mmol/l (it does not go higher precisely because the CNS efficiently uses these molecules for energy in place of glucose) and with no change in pH, whereas in uncontrolled diabetic ketoacidosis it can exceed 20 mmol/l with a concomitant lowering of blood pH^9, 10^ (Table 1).

## Therapeutic roles of ketogenic diets

### Strong evidence

#### Weight loss

There is no doubt that there is strong supportive evidence that the use of ketogenic diets in weight-loss therapy is effective; however, there are contrasting theories regarding the mechanisms through which they work. Some researchers suggest that there are not in fact any metabolic advantages in low-carbohydrate diets and that weight loss results simply from reduced caloric intake, probably due to the increased satiety effect of protein.^12^ Others instead promote the hypothesis that there is indeed a distinct metabolic advantage, which has recently been explored in more detail, raising interest in the role of VLCKD in weight loss and effects on metabolism in general.^13^ The first law of thermodynamics, also known as the law of conservation of energy, has in effect controlled the concepts for the basis of weight loss for over a century—resulting in a difficulty in accepting other ways of thinking. Adhering to these traditional concepts the US Department of Agriculture has concluded that diets, which reduce calories, will result in effective weight loss independent of the macronutrient composition, which is considered less important, even irrelevant.^14^ In contrast with these views, the majority of *ad-libitum* studies demonstrate that subjects who follow a low-carbohydrate diet lose more weight during the first 3–6 months compared with those who follow balanced diets.^15, 16, 17^ One hypothesis is that the use of energy from proteins in VLCKD is an ‘expensive' process for the body and so can lead to a ‘waste of calories', and therefore increased weight loss compared with other ‘less-expensive' diets.^13, 18, 19^ The average human body requires 60–65 g of glucose per day, and during the first phase of a diet very low in carbohydrates this is partially (16%) obtained from glycerol, with the major part derived via gluconeogenesis from proteins of either dietary or tissue origin.^12^ The energy cost of gluconeogenesis has been confirmed in several studies^7^ and it has been calculated at ∼400–600 Kcal/day (due to both endogenous and food source proteins.^18^ Despite this, there is no direct experimental evidence to support this intriguing hypothesis; on the contrary, a recent study reported that there were no changes in resting energy expenditure after a VLCKD.^20^ A simpler, perhaps more likely, explanation for improved weight loss is a possible appetite-suppressant action of ketosis. The mechanism for this is not established but evidence supports direct action of KBs together with modifications in levels of hormones, which influence appetite, such as ghrelin and leptin.^21^ Here we can summarize (listed in order of importance and available evidence) that the weight-loss effect of VLCKD seems to be caused by several factors:

1. Reduction in appetite due to higher satiety effect of proteins,^12, 22^ effects on appetite control hormones^21^ and to a possible direct appetite-suppressant action of the KBs.^23^
2. Reduction in lipogenesis and increased lipolysis.^7, 10^
3. Reduction in the resting respiratory quotient and, therefore, greater metabolic efficiency in consuming fats.^20, 24^
4. Increased metabolic costs of gluconeogenesis and the thermic effect of proteins.^13, 18^

#### Cardiovascular disease

Several lines of evidence point to beneficial effects of VLCKD on cardiovascular risk factors. In the past, there have been doubts expressed about their long-term safety and increased effectiveness compared with ‘balanced' diets,^25^ and clearly negative opinions regarding possible deleterious effects on triglycerides and cholesterol levels in the blood.^26^ However, the majority of recent studies seem instead to amply demonstrate that the reduction of carbohydrates to levels that induce physiological ketosis (see above ‘What is ketosis?' section) can actually lead to significant benefits in blood lipid profiles.^15, 17, 27^ The VLCKD effect seems to be particularly marked on the level of blood triglycerides,^24, 28^ but there are also significant positive effects on total cholesterol reduction and increases in high-density lipoprotein.^24, 28, 29^ Furthermore, VLCKD have been reported to increase the size and volume of low-density lipoprotein–cholesterol particles,^29^ which is considered to reduce cardiovascular disease risk, as smaller low-density lipoprotein particles have a higher atherogenicity. There are also direct diet-related effects on overall endogenous cholesterol synthesis. A key enzyme in cholesterol biosynthesis is 3-hydroxy-3-methylglutaryl–CoA reductase (the target for statins), which is activated by insulin, which means that an increase in blood glucose and consequently of insulin levels will lead to increased endogenous cholesterol synthesis. A reduction in dietary carbohydrate will have the opposite effect and this, coupled with the additional inhibition by dietary cholesterol and fats on endogenous synthesis, is likely to be the mechanism via which physiological ketosis can improve lipid profiles. Hence, there are strong doubts about the negative effects of dietary fats when they are consumed as part of a VLCKD, on cholesterol and triglycerides blood levels, whereas there are strong pointers to the beneficial effects of VLCKD on these cardiovascular risk parameters.^27, 28^

#### Type 2 diabetes

Insulin resistance is the primary feature underlying type 2 diabetes (T2D) but it also exists across a continuum in the general population, and to varying extents it disrupts insulin action in cells, which can cause a wide spectrum of signs and symptoms. A primary feature of insulin resistance is an impaired ability of muscle cells to take up circulating glucose. A person with insulin resistance will divert a greater proportion of dietary carbohydrate to the liver where much of it is converted to fat (that is, *de novo* lipogenesis), as opposed to being oxidized for energy in skeletal muscle.^30^ Although Hellerstein^31^ has recently reported that *de novo* lipogenesis contributes only ∼20% of new triglycerides, this greater conversion of dietary carbohydrate into fat, much of it entering the circulation as saturated fat, is a metabolic abnormality that significantly increases risk for diabetes and heart disease. Thus, insulin resistance functionally manifests itself as ‘carbohydrate intolerance'. When dietary carbohydrate is restricted to a level below which it is not significantly converted to fat (a threshold that varies from person to person), signs and symptoms of insulin resistance improve or often disappear completely.

In studies that have evaluated well-formulated very-low-carbohydrate diets and documented high rates of compliance in individuals with T2D, results have been nothing short of remarkable. Bistrian *et al.*^32^ reported withdrawal of insulin and major weight loss in a matter of weeks in T2D individuals who were fed a very-low-calorie and -carbohydrate diet. Gumbiner *et al.*^33^ fed obese T2D individuals two types of hypocaloric (650 kcal) diets for 3 weeks, they were matched for protein but one was much lower in carbohydrate content (24 vs 94 g/day). As expected, the lower-carbohydrate diet resulted in significantly greater levels of circulating ketones (∼3 mmol/l), which was strongly associated with a lower hepatic glucose output. Interestingly, there was a strong inverse correlation between circulating ketones and hepatic glucose output, suggesting that higher levels of ketones are associated with more favourable effects on glycaemic control in diabetics. More recently, Boden *et al.*^34^ performed an in-patient study in obese T2D individuals who were fed a low-carbohydrate (<20 g/day) diet for 2 weeks. Plasma glucose fell from 7.5 to 6.3 mmol/l, haemoglobin A1c decreased from 7.3 to 6.8% and there were dramatic improvements (75%) in insulin sensitivity.

In a longer study^35^ obese T2D individuals were prescribed a well-formulated ketogenic diet for 56 weeks, and significant improvements in both weight loss and metabolic parameters were seen at 12 weeks and continued throughout the 56 weeks as evidenced by improvements in fasting circulating levels of glucose (−51%), total cholesterol (−29%), high-density lipoprotein–cholesterol (63%), low-density lipoprotein–cholesterol (−33%) and triglycerides (−41%). It is of interest to note that in a recent study in overweight non/diabetic subjects, it was reported that during ketosis fasting glucose was not affected, but there was an elevation in post-prandial blood glucose concentration. This data suggests a different effect of ketosis on glucose homeostasis in diabetic and non-diabetic individuals.^21^ Other studies support the long-term efficacy of ketogenic diets in managing complications of T2D.^36, 37^ Although significant reductions in fat mass often results when individuals restrict carbohydrate, the improvements in glycaemic control, haemoglobin A1c and lipid markers, as well as reduced use or withdrawal of insulin and other medications in many cases, occurs before significant weight loss occurs. Moreover, in isocaloric experiments individuals with insulin resistance showed dramatically improved markers of metabolic syndrome than diets lower in fat.^27^ It is interesting in this respect that a recent extremely large epidemiological study reported that diabetes risk is directly correlated, in an apparently causative manner, with sugar intake alone, independently of weight or sedentary lifestyle.^38^

In summary, individuals with metabolic syndrome, insulin resistance and T2D (all diseases of carbohydrate intolerance) are likely to see symptomatic as well as objective improvements in biomarkers of disease risk if they follow a well-formulated very-low-carbohydrate diet. Glucose control improves not only because there is less glucose coming in, but also because systemic insulin sensitivity improves as well.

#### Epilepsy

Since 1920, the ketogenic diet has been recognized as an effective tool in the treatment of severe childhood epilepsy, but following the introduction of anticonvulsant drugs, the interest in ketogenic diet treatment waned until the 1990s, with subsequent research and clinical trials demonstrating its practical usefulness. Various studies have been carried out to understand its mechanism of action in epilepsy, but until now it remains largely uncertain.^5^ Several hypotheses have been put forward trying to explain the mechanism of action of ketogenic diets: (1) a direct anticonvulsant effect of KBs; (2) a reduced neuronal excitability induced by KBs;^39^ (3) an effect on the mammalian target of rapamycin pathway.^40^ In 2008, Hartman *et al.*^41^ demonstrated the efficacy of a ketogenic diet in the 6-Hz seizure test in mice, but also reported that the protection from seizures was not related to the level of ketosis in the blood, indicating that mechanism(s) of action other than those directly linked to the blood concentration of KBs should be investigated. Most researchers suggest that the metabolic mechanism(s) activated by ketogenic diets (see above) may influence neurotransmitter activity in neurons and this is currently a field of active research. Although the mechanisms of action are not clear, the ketogenic diet is now considered an established part of an integrative approach, along with drug therapy, in the major epilepsy centres worldwide,^42^ an important benefit being the reduction of drug use and concomitant reductions in severe side effects often associated with antiepileptic agents. The effectiveness of ketogenic diets is strongly supported in a recent Cochrane review where all studies showed a 30–40% reduction in seizures compared with comparative controls, and the review authors reported that in children the effects were ‘comparable to modern antiepileptic drugs'. The main drawback with the ketogenic diet was difficult tolerability and high dropout rates—given the extremely positive results and the severe side effects common with antiepilepsy medication, the development of an easier-to-follow ketogenic diet would be a worthwhile goal.^5^

**Figure 1:**
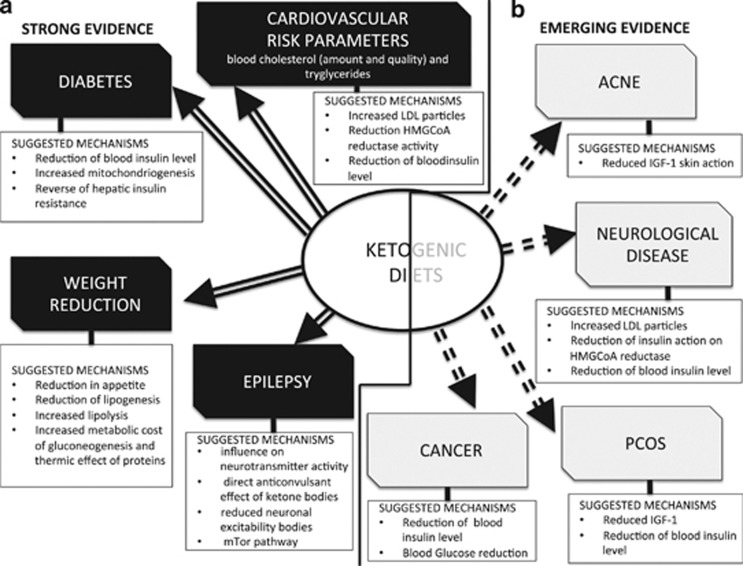
Suggested mechanisms for the therapeutic action of ketogenic diets in pathologies for which there exists strong (**a**) and emerging (**b**) evidence.

In conclusion, the role of ketogenic diets in epilepsy treatment is well established and we are confident that this is also the case for weight loss, cardiovascular disease and T2D. The recent research reviewed here demonstrate improvements in many risk factors, such as weight, saturated fats, inflammation and other biomarkers, as a consequence of consuming well-formulated low-carbohydrate diets, and this work should encourage continued close examination of their therapeutic value (Figure 1).

### Emerging evidence

#### Acne

In recent years there have been an increasing number of studies published, suggesting that at least for certain food types there is a nutritional influence on the development of acne. The negative effects seem to lie in the capacity of some foods/nutrients to stimulate proliferative pathways that in turn stimulate development of acne—suspect foods include those with a high glycaemic load and milk.^11, 43, 44^ Other evidence comes from several studies reporting that the prevalence of acne varies significantly between different populations and is substantially lower in non-Westernized populations that follow traditional diets,^45^ a common factor among these traditional diets being a low glycaemic load.^46^ Various studies have provided evidence that high-glycaemic-load diets are implicated in the aetiology of acne through their capacity to stimulate insulin, androgen bioavailability and insulin-like growth factor-1 (IGF-1) activity, whereas the beneficial effects of low-glycaemic-load diets, apart from weight and blood glucose levels, also include improved skin quality.^44^ The clinical and experimental evidence does in fact suggest ways in which insulin can increase androgen production and affect via induction of steroidogenic enzymes,^47^ the secretion by the pituitary gland of gonadotropin-releasing hormone and the production of sex hormone-binding globulin.^48^ Insulin is also able to reduce serum levels of IGF-binding protein-1 increasing the effect of IGF-1.^49^ These insulin-mediated actions can therefore influence diverse factors that underlie the development of acne such as:

- The increased proliferation of basal keratinocytes within the pilosebaceous ducts.
- An abnormal desquamation of the follicular epithelium.
- Increased androgen-mediated sebum production.
- Colonization of the stratum corneum by *Propionibacterium acnes* and consequent inflammation.^46^

In summary, there is persuasive, although not yet conclusive, clinical and physiological evidence that the ketogenic diet could be effective in reducing the severity and progression of acne and randomized clinical trials will be required to resolve the issue.^11^

#### Cancer

Carcinogenesis is a complex process involving multiple sequential mutations, which occur randomly in the DNA of normal cells over many years, even decades, until finally specific genes are mutated and cell growth becomes out of control resulting in the full neoplastic phenotype and often metastasis. There is evidence that hyperinsulinaemia, hyperglycaemia and chronic inflammation may affect the neoplastic process through various pathways, including the insulin/IGF-1 pathway, and most cancer cells express insulin and IGF-1 receptors. Insulin has been shown to stimulate mitogenesis (even in cells lacking IGF-1 receptors)^50^ and it may also contribute by stimulating multiple cancer mechanisms, including proliferation, protection from apoptotic stimuli, invasion and metastasis.^51^ The IFG1/insulin pathway may also enhance the promotion and progression of many types of cancer cells and facilitate cancer diffusion through angiogenesis.^52^ Insulin may act directly, but also indirectly through IGF-1, as it reduces hepatic IGF-binding protein-1 and -2 production,^53^ thereby increasing the levels of circulating, free active IGF-1, which may have a role in cancerogenesis due to its mitogenic and antiapoptotic activity.^53^ Considering the obvious relationship between carbohydrates and insulin (and IGF-1) a connection between carbohydrate and cancer is a possible consequence, and some links have been recognized since the 1920s when the Russo-German physician Dr A Braunstein observed that glycosuria falls off notably in diabetic patients who developed cancer.^54^ Later Warburg *et al.*^55^ of the Kaiser Wilheim Institute fur biologie described what was later known as the Warburg effect—where energy is predominantly generated by a high rate of glycolysis followed by lactic acid fermentation in the cytosol, even in the presence of plentiful oxygen.^51, 55^ The Warburg effect has been confirmed in many studies and today is a well-established hallmark of many types of cancers, and rapidly growing tumour cells typically have glycolytic rates up to 200 times higher than those of their normal tissues of origin.^56^ As stated above, the stimulus of the insulin/IGF-1 pathway is involved in cancer development, but also mitochondrial damage or dysfunction may have a role.^18, 57, 58^ Dysfunctional mitochondria may upregulate some oncogenes of the phosphatidylinositol 3-kinase/Akt mammalian target of rapamycin signalling pathway.^58^ Akt, a downstream of insulin signalling,^59^ is involved in changes in tumour cell metabolism and increases resistance to apoptosis; it also decreases β-oxidation and increases lipid synthesis in the cytosol.^60^ Hence, it seems a reasonable possibility that a very-low-carbohydrate diet could help to reduce the progression of some types of cancer, although at present the evidence is preliminary.^61^ In the 1980s, seminal animal studies by Tisdale and colleagues^62, 63^ demonstrated that a ketogenic diet was capable to reduce tumour size in mice, whereas more recent research has provided evidence that ketogenic diets may reduce tumour progression in humans, at least as far as gastric and brain cancers are concerned.^64, 65, 66, 67^ Although no randomized controlled trials with VLCKD have yet been conducted on patients and the bulk of evidence in relation to the influence of VLCKD on patient survival is still anecdotal,^68, 69, 70^ a very recent paper by Fine *et al.*^71^ suggests that the insulin inhibition caused by a ketogenic diet could be a feasible adjunctive treatment for patients with cancer. In summary, perhaps through glucose ‘starvation' of tumour cells and by reducing the effect of direct insulin-related actions on cell growth, ketogenic diets show promise as an aid in at least some kind of cancer therapy and is deserving of further and deeper investigation—certainly the evidence justifies setting up clinical trials.

#### Polycystic ovary syndrome

Polycystic ovary syndrome (PCOS) is a common endocrine disorder in females, with a high prevalence (6–10%);^72^ symptoms include hyperandrogenism, ovulatory dysfunction, obesity, insulin resistance and subfertility. Insulin resistance and related hyperinsulinaemia is actually a very common feature affecting about 65–70% of women with PCOS;^73^ it is seen most frequently in obese patients, affecting 70–80%, compared with only 20–25% of lean PCOS sufferers.^72^ Despite this observation, insulin resistance and hyperinsulinaemia appear to be linked to PCOS independently of obesity, and modifications in the normal cellular mechanisms of insulin signalling have been demonstrated in both lean and obese patients. Furthermore, high blood levels of insulin can act by increasing androgenous hormonal stimulation of the ovarian theca cells as well as potentiating gonadotropin-stimulated ovarian androgen steroidogenesis—although recent data has suggested that the insulin-induced increase in ovarian hormone secretion is not accompanied by increased steroid metabolism.^74^ Hyperinsulinaemia may also affect the central actions of androgen by impairing progesterone inhibition of the gonadotropin-releasing hormone pulse generator.^75^ Insulin has also been shown to increase expression of adrenal steroidogenic enzyme mRNA^47^ as well as adrenal responsiveness to adrenocorticotropic hormone.^76^

Women with PCOS frequently demonstrate many of the signs related to metabolic syndrome, such as insulin resistance, obesity, glucose intolerance, T2D, dyslipidemia and also high levels of inflammation. Suggested treatments include those that reduce insulin resistance/hyperinsulinaemia, such as lifestyle modifications (exercise, diet and weight loss) and pharmacological treatments that include the administration of thiazolidinediones or metformin. It is evident that any interventions that improve insulinaemia and reduce body weight may also be effective in reducing hyperandrogenism, normalizing ovulation and reducing the various symptoms of PCOS.

Finally, although we only have preliminary evidence of the positive effects of VLCKD in PCOS,^77^ there are clear mechanisms that are consistent with the physiological plausibility of such dietary therapy.

#### Neurological diseases

Emerging data suggest a possible therapeutic utilization of ketogenic diets in multiple neurological disorders apart from epilepsy,^78^ including head ache, neurotrauma, Alzheimer's and Parkinson's disease, sleep disorders, brain cancer, autism and multiple sclerosis.^79^ Although these various diseases are clearly different from each other, a common basis potentially explaining ketogenic diet efficacy could be a neuroprotective effect in any disease in which the pathogenesis includes abnormalities in cellular energy utilization, which is a common characteristic in many neurological disorders.^79^ The exact mechanism(s) by which a ketogenic diet may act is still poorly understood; however, some published reports can provide useful suggestions. For example, KBs were recently reported to act as neuroprotective agents by raising ATP levels and reducing the production of reactive oxygen species in neurological tissues,^80^ together with increased mitochondrial biogenesis, which may help to enhance the regulation of synaptic function.^80^ Moreover, the increased synthesis of polyunsaturated fatty acids stimulated by a KD may have a role in the regulation of neuronal membrane excitability: it has been demonstrated, for example, that polyunsaturated fatty acids modulate the excitability of neurons by blocking voltage-gated sodium channels.^81^ Another possibility is that by reducing glucose metabolism, ketogenic diets may activate anticonvulsant mechanisms, as has been reported in a rat model.^82^ In addition, caloric restriction per se has been suggested to exert neuroprotective effects, including improved mitochondrial function, decreased oxidative stress and apoptosis, and inhibition of proinflammatory mediators, such as the cytokines tumour necrosis factor-α and interleukins.^83^ Although promising data have been collected (see below), at the present time the real clinical benefits of ketogenic diets in most neurological diseases remain largely speculative and uncertain, with the significant exception of its use in the treatment of convulsion diseases.

#### Alzheimer's disease

Patients affected with Alzheimer's disease show a higher incidence of seizures compared with unaffected people,^84^ and it has recently been reported that neuronal excitability is enhanced,^85, 86^ and neuronal circuits and mitochondrial homeostasis are altered.^87^

On the basis of the reports described above, these results indicate a possible role of the ketogenic diet in the treatment of Alzheimer's disease in the clinic. Supporting evidence is provided by a study, which reported that at least in selected conditions a significant clinical improvement was observed in Alzheimer's patients fed a ketogenic diet.^88^ It was suggested that this was, at least in part, related to improved mitochondrial function secondary to the reported protective effects of KBs against the toxic consequences of the exposure of cultured neurons to β-amyloid.^89^ In an animal model of Alzheimer's disease, transgenic mice consuming a ketogenic diet exhibited better mitochondrial function and less oxidative stress and β-amyloid deposition when compared with normally fed controls.^90^ These promising results should encourage further research that is necessary to improve our understanding about the potential benefits of ketogenic diets in this debilitating and, thus far, irreversible disease.

#### Parkinson's disease

The possible beneficial effects of ketogenic diets on mitochondrial activity described above has also been proposed to explain the improved scores on a standard gravity scale of Parkinson' disease exhibited by some patients.^91^ In addition, the typical mitochondrial respiratory chain damage that occurs in animal models of Parkinson's disease was reduced by a ketogenic diet;^89^ however, the real utility of this diet remains largely speculative and uncertain.

#### Brain trauma

Traumatic brain injury may lead over time to epilepsy. Because of the effective use of the ketogenic diet in reducing seizures (see above), it has been suggested that it may also improve the clinical status in brain injury, especially by reducing the incidence of long-term consequences, such as epilepsy.^79^ Positive effects of a ketogenic diet have also been reported in reducing the cortical contusion volume in an age-dependent manner in an animal model of cortical injury, which is related to the maturation-dependent variability in brain ketone metabolism.^92^ These findings were also supported by the demonstration that a ketogenic diet reduced post-traumatic cognitive and motor function impairment, at least in a rat model.^93^

The antiepileptogenic activity of the ketogenic diet after traumatic brain damage is controversial though,^94^ and further studies are needed to increase related knowledge.

#### Amyotrophic lateral sclerosis

Dysfunction in energy production, that is, mitochondrial function impairment, is likely to have a role in the pathogenesis of many neurodegenerative diseases, perhaps including amyotrophic lateral sclerosis. On this basis, a ketogenic diet has been proposed as a collateral therapeutic approach in this disease.^95^ Studies by Zhao *et al.*^96^ revealed both histological and functional improvements in an animal model of amyotrophic lateral sclerosis when a ketogenic diet was given compared with when given a control diet. Although survival time was not increased, a higher motor neuron count and lower motor function impairment was reported among the findings.

Nevertheless, direct experimentation and clinical trials in humans are still lacking at the present time, and to avoid the possibility of generating false hopes the preliminary data from animal models obviously have to be considered very cautiously.

#### The effect of a ketogenic diet on respiratory function

The metabolic effects of a ketogenic diet imply a higher-than-usual oxidation of fats, which leads in turn to reduced respiratory exchange ratio values.^20, 97^ Metabolic carbon dioxide output may be calculated as the product of alveolar ventilation multiplied by the fractional alveolar carbon dioxide concentration. Pulmonary ventilation differs from alveolar ventilation only by the amount of physiological dead space, and there is no reason to suspect a change in physiological dead space when a dietary manipulation is applied. Hence, following a ketogenic diet-induced decrement of the respiratory exchange ratio and of metabolic carbon dioxide output, a decrease in arterial carbon dioxide partial pressure or of pulmonary ventilation, or of both, is expected. If verified, these effects might be useful in the treatment of patients with respiratory failure; however, this aspect of the ketogenic diet remains to be investigated. Sabapathy *et al.*^98^ observed that the reduction in muscle glycogen content caused a respiratory exchange ratio decrement, which was associated with reduced carbon dioxide partial pressure and constancy of pulmonary ventilation. These findings at least suggest potential useful effects of this diet in patients with increased carbon dioxide, arterial partial-pressure values as a consequence of respiratory failure. Of course, more studies are needed to verify this working hypothesis.

#### Potential risks of ketogenic diets

If we equate *de facto* ketogenic diets with high-protein diets (which is not always correct) then the risks proposed by critics of this type of dietary approach are essentially those of possible kidney damage due to high levels of nitrogen excretion during protein metabolism, which can cause an increase in glomerular pressure and hyperfiltration.^12^ There is not wide agreement between studies; however, some infer the possibility of renal damage from animal studies,^99, 100^ whereas others, looking at both animal models, meta-analyses and human studies, propose that even high levels of protein in the diet do not damage renal function.^101, 102^ In subjects with intact renal function, higher dietary protein levels caused some functional and morphological adaptations without negative effects.^103^ There may actually be renal-related effects, but on blood pressure rather than morphological damage. The amino acids involved in gluconeogenesis and/or production of urea in general have blood-pressure-lowering effects, whereas acidifying amino acids tend to cause a rise in blood pressure. Subjects with renal insufficiency, even subclinical, kidney transplant patients and people with metabolic syndrome or other obesity-related conditions, will be more susceptible to the hypertensive effect of amino acids, especially of the sulphated variety.^104^ The well-documented correlation between obesity and reduced nephron quantity on raised blood pressure puts subjects with T2D or metabolic syndrome at risk, even if in diabetics with kidney damage the effects are not always consistent with the hypothesis.^12,105,106^ In fact, although some authors have reported a positive influence of a reduction in protein intake from 1.2 to 0.9 g/kg, over the short term, on albuminuria in T2D,^107^ the same authors have subsequently stated instead that dietary protein restriction is neither necessary nor useful over the long term.^108^

Moreover, it should be noted that ketogenic diets are only relatively high in protein^18, 106^ and that some recent studies have demonstrated that VLCKD can even cause a regression of diabetic nephropathy in mice.^109^ With regard to possible acidosis during VLCKD, as the concentration of KBs never rises above 8 mmol/l^10^ this risk is virtually inexistent in subjects with normal insulin function.

## Conclusions

Ketogenic diets are commonly considered to be a useful tool for weight control and many studies suggest that they could be more efficient than low-fat diets, although there is not concordance in the literature about their absolute effectiveness and even some doubts raised about safety. But there is a ‘hidden face' of the ketogenic diet: its broader therapeutic action. There are new and exciting scenarios about the use of ketogenic diets, as discussed in this review, in cancer, T2D, PCOS, cardiovascular and neurological diseases. Further studies are warranted to investigate more in detail the potential therapeutic mechanisms, its effectiveness and safety, and we would invite all researchers to face this challenge without prejudice.

## Figures and Tables

**Table 1:** Blood levels during a normal diet, ketogenic diet and diabetic ketoacidosis^11^

*Blood levels*	*Normal diet*	*Ketogenic diet*	*Diabetic ketoacidosis*
Glucose (mg/dl)	80–120	65–80	>300
Insulin (μU/l)	6–23	6.6–9.4	≅ 0
KB conc (mℳ/l)	0.1	7/8	>25
pH	7.4	7.4	<7.3
